# Supplementary material for: Identification of photoperiod-induced specific miRNAs in the adrenal glands of Sunite sheep (Ovis aries)
Source: Front Vet Sci. 2022 Jul 22;9:888207. doi: 10.3389/fvets.2022.888207 (PMC9354845; doi:10.3389/fvets.2022.888207)
Supplement: Supplementary file 1 [file Data_Sheet_1.ZIP › Supplementary file/miRDeep2 file.zip!/miRDeep2 file/Novel_Structure/SPLP42A1/Novel_111.pdf]

Provisional ID	:	NC_019458.2_787
Score total	:	0.2
Score for star read(s)	:	-1.3
Score for read counts	:	0
Score for mfe	:	0
Score for randfold	:	1.6
Score for cons. seed	:	
Total read count	:	117
Mature read count	:	117
Loop read count	:	0
Star read count	:	0

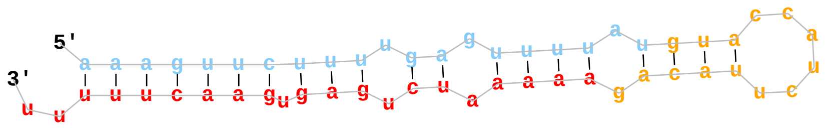

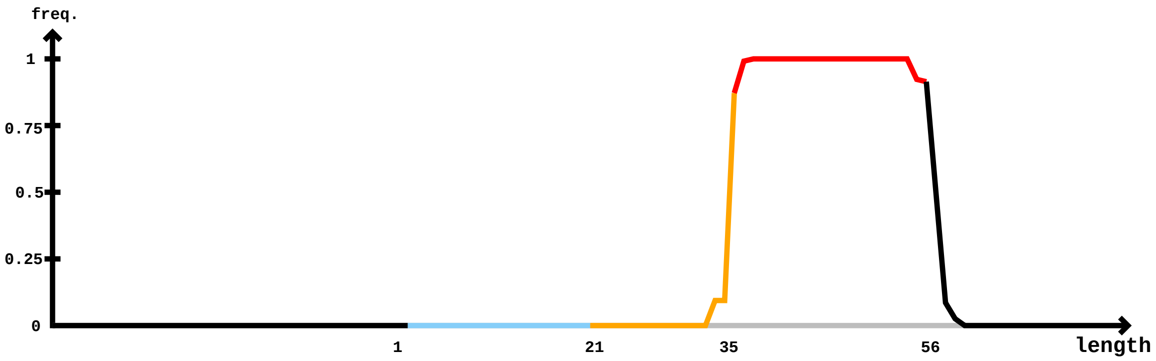

Star Mature

```
5'- auauuuuuauuuguaacuaaguaugggcugaaaaaaaaguucuuuugaguuuuauguaccaucuuacagaaaaaucugagugaacuuuuuggccagcccaguauuaaaua   -3'    exp
    .................(((...(((((((...((((((((((((.((.((((.((((......)))).)))).)).))).)))))))))...)))))))...)))....    reads       mm         sample
    ...................................................................agaaaaaucugaAugaacuuuuu....................    11          1          seq
    .....................................................................aaaaaucugaAugaacuuu......................    9           1          seq
    .....................................................................aaaaaucugagugaacuuuu.....................    1           0          seq
    .....................................................................aaaaaCcugagugaacuuuuu....................    1           1          seq
    .....................................................................aaaaaucugaAugaacuuuuu....................    80          1          seq
    ......................................................................aaaaucugagugaacuuuuu....................    5           0          seq
    ......................................................................aaaaucugagugaacuuuuugg..................    6           0          seq
    ......................................................................aaaaucugagugaacuuuuuggc.................    1           0          seq
    ......................................................................aaaaucugaAugaacuuuuuggc.................    2           1          seq
    .......................................................................aaaucugagugaacuuuuugg..................    1           0          seq
```
